# Biomass allometric models for *Larix rupprechtii* based on Kosak’s taper curve equations and nonlinear seemingly unrelated regression

**DOI:** 10.3389/fpls.2022.1056837

**Published:** 2023-01-09

**Authors:** Dongzhi Wang, Zhidong Zhang, Dongyan Zhang, Xuanrui Huang

**Affiliations:** ^1^ College of Forestry, Hebei Agricultural University, Baoding, China; ^2^ College of Economics and Management, Hebei Agricultural University, Baoding, China

**Keywords:** allometric models, universal scaling, taper equations, nonlinear regression, nonlinear seemingly unrelated regression

## Abstract

The diameter at breast height (DBH) is the most important independent variable in biomass allometry models based on metabolic scaling theory (MST) or geometric theory. However, the fixed position DBH can be misleading in its use of universal scaling laws and lead to some deviation for the biomass model. Therefore, it is still an urgent scientific problem to build a high-precision biomass model system. A dataset of 114 trees was destructively sampled to obtain dry biomass components, including stems, branches, and foliage, and taper measurements to explore the applicability of biomass components to allometric scaling laws and develop a new system of additive models with the diameter in relative height (DRH) for each component of a Larch (*Larix principis-rupprechtii* Mayr) plantation in northern China. The variable exponential taper equations were modelled using nonlinear regression. In addition, applying nonlinear regression and nonlinear seemingly unrelated regression (NSUR) enabled the development of biomass allometric models and the system of additive models with DRH for each component. The results showed that the Kozak’s (II) 2004 variable exponential taper equation could accurately describe the stem shape and diameter in any height of stem. When the diameters in relative height were D_0.2_, D_0.5_, and D_0.5_ for branches, stems, and foliage, respectively, the allometric exponent of the stems and branches was the closest to the scaling relations predicted by the MST, and the allometric exponent of foliage was the most closely related to the scaling relations predicted by geometry theory. Compared with the nonlinear regression, the parameters of biomass components estimated by NSUR were lower, and it was close to the theoretical value and the most precise at forecasting. In the study of biomass process modelling, utilizing the DRH by a variable exponential taper equation can confirm the general biological significance more than the DBH of a fixed position.

## 1 Introduction

With the rapid changes in the global climate (Trumbore et al., 2015; Seidl et al., 2017), global warming of 1.5°C will have a dangerous and irreversible impact on the structure and function of countries (Hoegh-Guldberg et al., 2019). As the primary part of the global carbon cycle, forests are highly significant at reducing greenhouse gas emissions, and thus, mitigating climate warming (Le Toan et al., 2011; Stephenson et al, 2014; Dai et al., 2021). The monitoring and evaluation of biomass plays a vital role in fully understanding the mechanism of contribution and predicting the potential of forests to serve as carbon sinks (Zeng and Tang, 2011; Zhu et al., 2018; Zhou et al., 2021).

In past few decades, many different forms of allometric equations (Ter-Mikaelian and Korzukhin, 1997; Muukkonen, 2007; Henry et al., 2011; Yuen et al., 2016) have been developed, and tree-level biomass models are the most accurate (Zeng et al., 2021). To date, an allometric relationship of the power exponent is the primary model used to estimate the biomass of a single tree, and these models are divided into process and empirical models (Fehrmann and Kleinn, 2006; Zianis et al., 2016). Unlike empirical model studies, a process model aims to explain the changes of proportion in tree structure with biological, physical, or mechanical factors and simulate its impact on the function and growth of different parts of a single tree (Fehrmann and Kleinn, 2006). Most biomass process models are based on metabolic scaling theory (MST) and geometric theory to reveal the allometric growth relationship between structural and functional characteristics within or between individual tree scales (West et al., 1997; Fournier et al., 2003; Návar, 2009). Based on the MST, West et al. (1999) showed that the theoretical value of allometric exponent was equal to 8/3 (≈2.67), which was closely related to the tree size and metabolic rate (Enquist et al., 2002; O’Connor and Bernhardt, 2018; Pettersen et al., 2019; Zhou et al., 2021). Nevertheless, based on a knowledge of geometry theory, the theoretical value of an allometric exponent can be deduced, i.e., the parameter value of biomass metabolic rate is close to 7/3 (≈2.33) (Fournier et al., 2003; Návar, 2009; Zeng and Tang, 2011). Zianis and Radoglou (2006) validated the MST model against 277 studies compiled and concluded that the theoretical average value of allometric exponent is statistically close to the geometric theoretical value but different from that of the MST (Zeng and Tang, 2011). To date, there is not enough evidence and methodology to draw a definite conclusion about whether the allometric exponent should be close to the fixed value of 8/3, 7/3 or other fractions for the biomass components of different tree species (West and Brown, 2005; LI et al, 2005; Reich et al, 2006).

Tree size is not only closely related to the theoretical value of allometric exponent of biomass component model but also to the determinant of its metabolic rate (West et al., 1997; Burger et al., 2019; Pettersen et al., 2019; Zhou et al., 2021). During the process of building a biomass model, the diameter at breast height (DBH) is an important index to reflect the size of trees (Enquist et al., 2002; O’Connor and Bernhardt, 2018). However, Fehrmann and Kleinn (2006) suggested that the use of DBH as an independent variable could mislead the universality of allometric exponent, and the relative increase in biomass was affected by the increase in relative diameter owing to the heterogeneity of stem shape (Cushman et al., 2014). The biomass process model based on a fixed position DBH leads to some deviation in the predictive effect of the model because the tree size and stem shape are variable (Fehrmann and Kleinn (2006); Cienciala et al., 2013). For trees with the same height and DBH, the tree that tapers less can have as much as 20% more volume (Heger, 1965; Klos et al., 2007), which would affect the accuracy of prediction using the biomass model. Under different stand conditions, the fixed position of DBH is not enough to explain the variation produced by the theoretical model of biomass metabolic rate of each organ (Cushman et al., 2014; Zheng et al., 2019; Forrester et al., 2021). Therefore, developing a new process model with the diameter in relative height (DRH) is an urgent problem for improving the accuracy of prediction and universality of the biomass model.

Trees are important carbon sinks and also Larch (*Larix principis-rupprechtii* Mayr) is one of the main afforestation tree species that has high social, economic and ecological value in northern China; it grows well and widely in warm temperate subalpine areas owing to its strong ability to adapt to the environment and the utility and performance of its wood (Qiu et al., 2019; Dong et al., 2019; Li et al., 2020). To achieve the goal of neutralizing global carbon and reducing its peak, accurate estimations of the biomass of *Larix principis-rupprechtii* are highly significant for the research on stand structure and function (Pan et al., 2013), evaluation of its production potential (Duan et al., 2018), and climate impact and adaptation (Rizvi et al., 2011; Zeng et al., 2021). However, a process model with the fixed position diameter (DBH) could mislead the universal scaling laws and lead to some deviation for the biomass model (Fehrmann and Kleinn (2006); Cienciala et al., 2013; Zianis et al., 2016). Therefore, constructing a biomass model using the DRH is critical to estimate, monitor, and evaluate the biomass and carbon sink of this larch.

Based on the biomass process model, the theoretical parameters of biomass component models for different tree species and the ability of DRH to meet the theoretical parameters have been researched less and remain controversial. Therefore, we propose a hypothesis that there is a relative position on the trunk whose diameter meets the theoretical value of biomass metabolic rate parameter and has the higher prediction accuracy for biomass components. To verify our hypothesis, *Larix principis-rupprechtii* plantation was taken as the research object, which enabled us to develop a model based on the study data of biomass components and taper measurements. 1) A taper equation was modeled to estimate the diameter at any height along the stem of a tree. 2) A theoretical model of biomass components was established using the DRH based on the nonlinear regression method, and 3) Nonlinear seemingly uncorrelated regression (NSUR) was applied to establish the additive model system of biomass components. A new additive model system of biomass components was also developed using the DRH to provide a scientific basis to improve the biomass and carbon storage of *Larix principis-rupprechtii* plantation.

## 2 Material and methods

### 2.1 Study area

The study area lies in the Saihanba Forest Farm (116°53’~118°31’ E, 41°22’~42°58’ N) in Hebei Province with a site type that is a warm temperate zone in north China. The altitude ranges from 1,012 m to 1,945 m, with an average annual temperature of -1.2°C. The average annual sunshine duration is 2,548.7 hours, with an annual maximum temperature and minimum temperature of 33.4 °C and - 43.3 °C, respectively. The average annual precipitation is approximately 452.2 mm, and the average annual evaporation is approximately 1 339.2 mm. The dominant forest types were *Larix principis-rupprechtii*, *Pinus sylvestris* var. *mongolica*, and *Picea asperata* plantations, and a *Betula platyphylla* secondary forest.

### 2.2 Data description

We used data from 114 sample plots that were established in the Saihanba Forest Farm of northern China. Each sample plot was 0.09 ha square (30 × 30 m^2^). The stand density ranges from 225 to 1 845 tree·hm^-2^ and the age distribution of stands ranges from 15 to 43 years. Within each plot, we measured every tree DBH (1.3 m above ground) ≥5 cm and height (H). According to the method of mean tree, one standard tree was selected for stem analysis in each sample plot. Comprehensive biomass and taper measurement datasets were collected from *Larix principis-rupprechtii* plantation by destructive sampling 114 trees. The stem was divided into 11 sections on the points of 0.00, 0.02, 0.04, 0.06, 0.08, 0.10, 0.15, 0.20, 0.30, 0.40, 0.50, 0.60, 0.70, 0.80, and 0.90 of relative tree height, and the diameters of all sections were measured (Zeng and Tang, 2011). In the field, the fresh weight of stem, branches and leaves was measured using the full weighing method. Moreover, samples of biomass components were selected, and their fresh weight was observed. All subsamples were oven-dried in the laboratory at 85°C until a constant weight was reached. We calculated the biomass of each component through its moisture content, and the biomass of the tree was obtained by summation. Summary statistics of the DBH, H, and biomass of different components are presented in **Table 1**.

**Table 1:** Summary statistics of tree variable measurements for the *Larix principis-rupprechtii* plantation biomass dataset in the study area.

Variables	Number of sample trees	Mean	Min	Max	SD	CV
DBH(cm)	114	17.4	5.1	26.8	5.6	32.3
D_i_(cm)	114	13.2	1.1	38.7	7.4	40.6
H(m)	114	13.6	3.5	21.9	4.2	30.9
Stem (kg)	114	85.4	2.37	256.2	64.8	75.8
Branches (kg)	114	21.1	1.14	58.2	14.9	70.7
Foliage (kg)	114	6.5	0.64	17.32	3.9	59.8
AGB (kg)	114	112.9	4.96	318.4	81.9	72.5

AGB, total tree aboveground biomass; CV, coefficient of variation; D_i_, diameter at relative tree height; SD, standard deviation.

### 2.3 Variable exponent taper model

Taper functions describe the law of variation in the stem shape and provide the diameter at any point along the stem (Menéndez-Miguélez et al., 2014; Liang et al., 2022). Cushman et al. (2014) found that the DRH predicted by the variable exponential taper equation was highly sensitive to the theoretical value of allometric exponent and could improve the accuracy of prediction of the process model. In recent decades, numerous taper equations, including simple taper functions, polynomial equations or segmented polynomial equations, and variable exponent taper functions, have been developed to predict the stem form (Kozak, 1988; Kozak, 2004; Shahzad et al., 2020). Compared with simple taper functions and segmented taper functions, variable exponent taper equations are more effective because they are highly flexible at predicting the stem diameter with minimum local deviation at any given height from the ground (Bi, 2000; Sharma and Oderwald, 2001; Jiang et al., 2005; Corral-Rivas et al., 2007; Lumbres et al., 2017). In many different forms of variable exponential taper equations, different forms of Kozak’s taper equations have been proven to be suitable for most tree species and are highly accurate at making predictions (Klos et al., 2007; Fonweban et al., 2012; He et al., 2021). Therefore, three forms of Kozak’s variable exponential taper equations were selected for our study.

The model form of Kozak (1988) is:


(1) $$d=b_{1}DBH^{b_{2}}b_{3}DBH{\left[\frac{1-\sqrt{T}}{1-\sqrt{0.01}}\right]}^{\left(b_{4}T^{2}{\left(\frac{1.3}{H}\right)}^{4}+b_{5}\ln(T+0.01)+b_{6}\sqrt{T}+b_{7}e^{T}+b_{8}\frac{DBH}{H}\right)}$$

The model form of Kozak (2004) is:


(2) $$d=b_{1}DBH^{b_{2}}{\left[\frac{1-T^{1/4}}{1-0.01^{1/4}}\right]}^{\left(b_{3}+b_{4}(\frac{1}{e^{DBH/H}})+b_{5}DBH(\frac{1-T^{1/4}}{1-0.01^{1/4}})+b_{6}{\left(\frac{1-T^{1/4}}{1-0.01^{1/4}}\right)}^{DBH/H}\right)}$$

The model form of Kozak (2004) is:


(3) d=b1DBHb2Hb3[1−T131−(1.3H)13]b4T4+b5(1eDBHH)+b6[1−T131−(1.3H)13]0.1+b7(1DBH)+b8H1−T13+b9(1−T131−(1.3H)13)

Where *d* is the predicted diameter at *h* (cm); *DBH* is the diameter at breast height (cm); *H* is the tree total height (m); *h* is the height from the ground (m), and *T*=*h*/*H*, *b_i_* are the estimated parameters. In equation 1, *b_3_* is a parameter related to DBH, and *b_4_* to *b_8_* are parameters related to DBH and tree height. In equation 2, *b_3_* is the intercept of the exponential part of the taper equation, and *b_4_* to *b_6_* are parameters related to DBH and tree height. In equation 3, *b_3_* is a parameter related to tree height, and *b_4_* to *b_9_* are parameters related to DBH and tree height.

### 2.4 Allometric models for the biomass component by nonlinear regression

The power exponent is the typical biomass allometric equation that has the characteristics of scale invariance (self-similarity) and universality (Marquet et al., 2005; Sileshi, 2014). However, the addition of variables or combinations of variables, such as crown variables, stand density, climate factors and age, can improve the accuracy of prediction of biomass models (Jucker et al., 2017; Forrester et al., 2021). Important to note that the linear or nonlinear biomass models based on multiple variables are neither based on any allometric growth theory nor follow the power-law function assumed by the typical allometric biomass model (Sileshi, 2014). The linear biomass allometric growth equation with logarithmic transformation cannot really solve the statistical problems related to the modeling of allometric growth relationship (Packard et al., 2010; Zianis et al., 2016). Henry et al. (2011) reported that 76% of the equations contain only one explanatory variable of DBH, but the DBH is insufficient to describe biomass allometric relationships (Ngomanda et al., 2014; Duncanson et al., 2015; Puc-Kauil et al., 2020). The allometric biomass model based on the DRH is more generally biologically significant than one based on the DBH (Djomo et al., 2010; Chave et al., 2014; Zianis et al., 2016). Thus, the following model was proposed to estimate the biomass for the *Larix principis-rupprechtii* plantation:


(4) $$W=aD_{i}^{\text{c}}+\epsilon$$

where *W* is the biomass of the tree component (stem, branch, and foliage), *D*_i_ is the diameter at relative tree height and the relative tree height is the ratio of height at a position to tree height, *a* is the estimated parameter, and *c* is the allometric exponent, *ϵ* is the error term for the tree component.

### 2.5 Nonlinear seemingly unrelated regression

The NSUR is an effective method (Bi and Long, 2001; Parresol, 2001; Cao, 2021) to ensure the additivity of biomass components and reduce the confidence and prediction intervals for the biomass (Dong et al., 2015). The independent variables of biomass equations were assumed to be fixed and observed without measurement errors (Fu et al., 2016). The NSUR of the *Larix principis-rupprechtii* plantation is described as follows:


(5) $$\left\{ \begin{array}{l} {\text{W}}_{\text{Stem}}={\text{a}}_{1}\text{Di}{\text{c}}_{1}+{\text{ϵ}}_{\text{Stem}}\\ {\text{W}}_{\text{Branch}}={\text{a}}_{2}\text{Di}{\text{c}}_{2}+{\text{ϵ}}_{\text{Branch}}\\ {\text{W}}_{\text{Foliage}}={\text{a}}_{3}\text{Di}{\text{c}}_{3}+{\text{ϵ}}_{\text{Foliage}}\\ {\text{W}}_{\text{AGB}}={\text{W}}_{\text{Stem}}+{\text{W}}_{\text{Branch}}+{\text{W}}_{\text{Foliage}}+{\text{ϵ}}_{\text{AGB}} \end{array}\right.$$

where *ϵ_Stem_*, *ϵ_Branch_*, *ϵ_Foliage_*, *ϵ_AGB_* are error terms; *D_i_* is the diameter at relative tree height, and *i* is the relative tree height. *a_1_, a_2_, a_3_, c_1_, c_2_, c_3_* are the parameters of biomass model. *W_stem_*, *W_branch_*, *W_foliage_* are the biomass of stems, branches, and foliage, and *W_AGB_* is the aboveground biomass.

### 2.6 Model fitting and evaluation

In our study, the parameter values of taper equations and biomass allometric equations were obtained using the PROC NLIN and PROC MODEL procedures of SAS version 9.4 (Littell et al., 2006). The accuracy and precision of taper equations and biomass allometric equations were assessed by the leave-one-out cross-validation approach (Timilsina and Staudhammer, 2013; Lehtonen et al., 2020). The goodness-of-fit was quantified by coefficient of determination (R^2^), the adjusted coefficient of determination (R_adj_^2^), mean absolute bias (MAB), mean percentage bias (MPB), and root mean square error (RMSE). The expressions for these statistics are as follows:


(6) $$R^{2}=1-\frac{\sum_{i=1}^{n}{\left(y_{i}-{\hat{y}}_{i}\right)}^{2}}{\sum_{i=1}^{n}{\left(y_{i}-{\bar{y}}_{i}\right)}^{2}}$$

(7) $$R_{\text{adj}}^{2}=1-(1-R^{2})(\frac{n-1}{n-\lambda })$$

(8) $$MAB=\frac{1}{n}\sum_{i=1}^{n}\left|y_{i}-{\hat{y}}_{i}\right|$$

(9) $$MPB=\frac{\sum_{i=1}^{n}\left|y_{i}-{\hat{y}}_{i}\right|}{\sum_{i=1}^{n}y_{i}}\times 100\%$$

(10) $$RMSE=\sqrt{\frac{1}{n-1}\sum_{i=1}^{n}{\left(y_{i}-{\hat{y}}_{i}\right)}^{2}}$$

where *y_i_*, *ŷ_i_*, and *ȳ_i_* are the observed, predicted, and average stem diameters or average biomass, respectively, in this study, and n is the total number of observed values, *λ* is the number of parameters.

## 3 Results

### 3.1 Taper equation fitting

**Figure 1:**
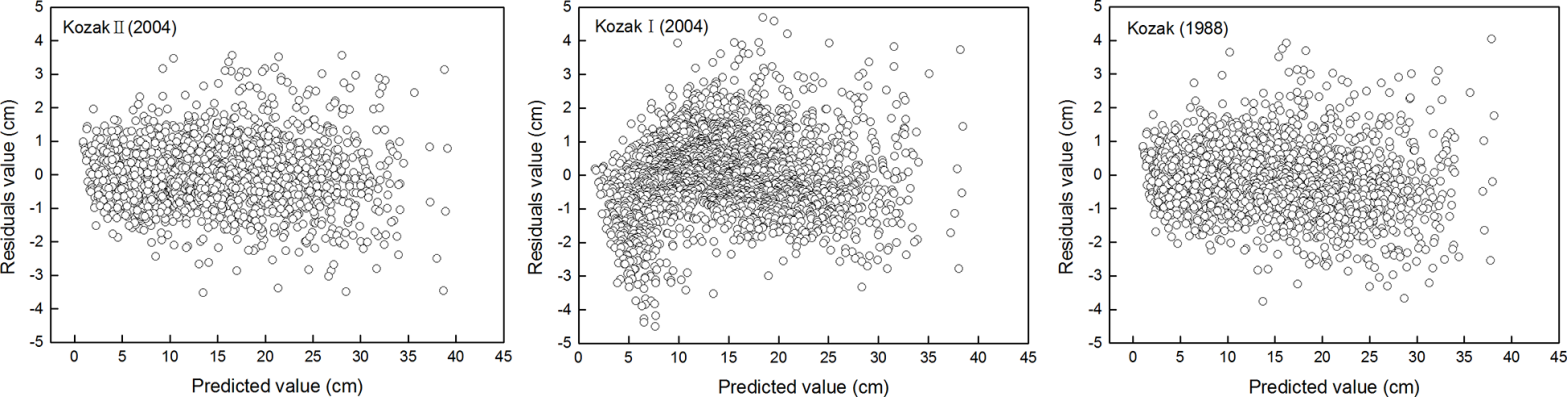
Residual analysis of three variable exponential taper equations for the *Larix principis-rupprechtii* plantation.

The parameter estimates and goodness-of-fit statistics of three different forms of the Kozak variable exponential taper equation are listed in **Tables 2** and **3**, respectively. Among all the variable exponential taper equations, the Kozak (2004) taper equation could accurately predict the diameter of different positions of the trunk. The adjusted coefficient of determination (R_adj_^2^), mean absolute bias (MAB), root mean square error (RMSE), and mean percentage of bias (MPB) of the equation were shown in **Table 3**. The standardized residual analysis of all the variable exponential taper equations is shown in **Figure 1**. The Kozak (2004) equation performed best, and no systematic trend in the distribution of residuals was observed.

**Table 2:** Parameter estimates for three forms of Kozak’s variable exponential taper equations.

Model	b_1_	b_2_	b_3_	b_4_	b_5_	b_6_	b_7_	b_8_	b_9_
Kozak (1988)	1.281 (0.050)	0.977 (0.022)	0.998 (0.001)	-1.002 (0.094)	0.235 (0.005)	-2.923 (0.083)	1.671 (0.032)	0.133 (0.012)	
KozakI(2004)	1.392 (0.025)	0.936 (0.005)	0.485 (0.001)	-0.137 (0.044)	0.003 (0.001)	-0.335 (0.015)			
KozakII (2004)	0.973 (0.015)	0.991 (0.008)	0.022 (0.009)	0.549 (0.015)	-1.035 (0.041)	0.668 (0.015)	1.265 (0.184)	0.010 (0.001)	-0.158 (0.013)

SEs (approximate standard errors) are in parentheses.

**Table 3:** Goodness-of-fit statistics of variable exponential taper equations using the cross-validation phase.

Model	MAB	RMSE	MPB	R_adj_^2^
Kozak (1988)	0.977	1.282	6.453	0.933
KozakI(2004)	0.785	0.994	4.923	0.950
KozakII (2004)	0.627	0.911	4.147	0.984

### 3.2 Allometric model development considering DRH

**Figure 2:**
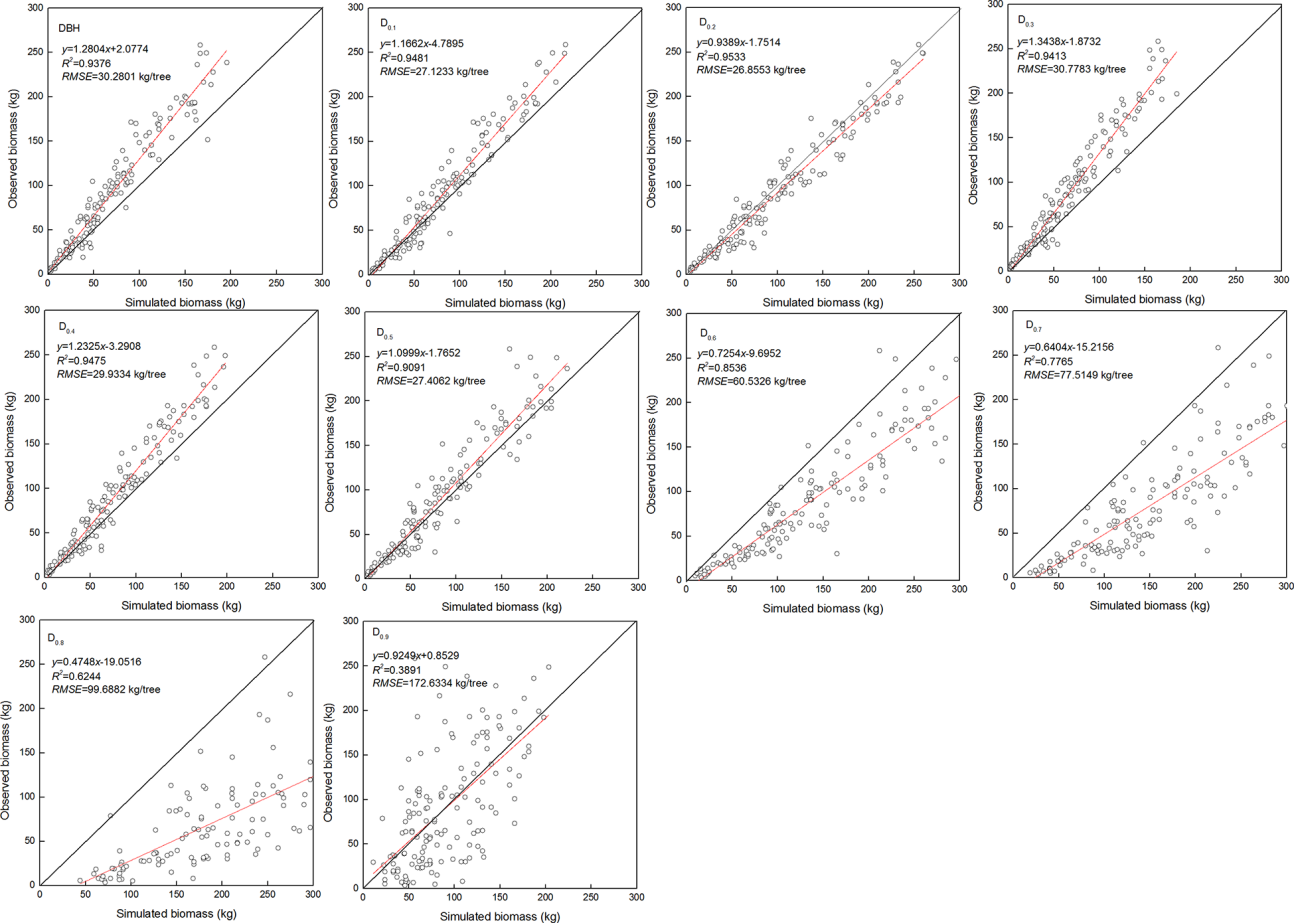
Linear relationship between observed and simulated data for stem biomass. Diameter at breast height (DBH), diameter at relative tree height (D_i_, i = 0.1 to 0.9), simulated values (y), observed values (x), coefficient of determination (R^2^), and root mean square error (RMSE). The black and red line represents the 1:1 isoline and linear equation, respectively.

**Figure 3:**
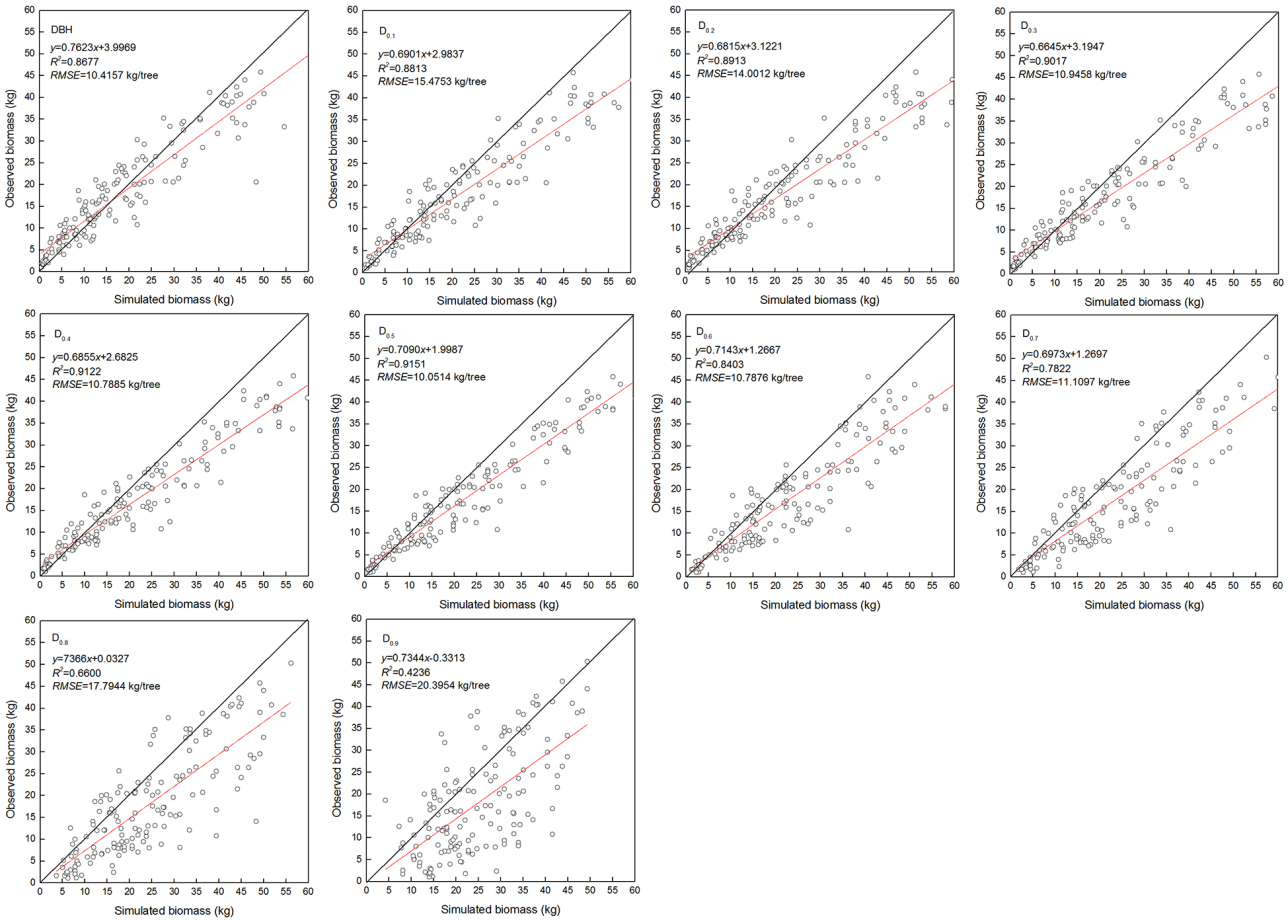
Linear relationship between observed and simulated data for branch biomass. Diameter at breast height (DBH), diameter at relative tree height (D_i_, i = 0.1 to 0.9), simulated values (y), observed values (x), coefficient of determination (R^2^), and root mean square error (RMSE). The black and red line represents the 1:1 isoline and linear equation, respectively.

**Figure 4:**
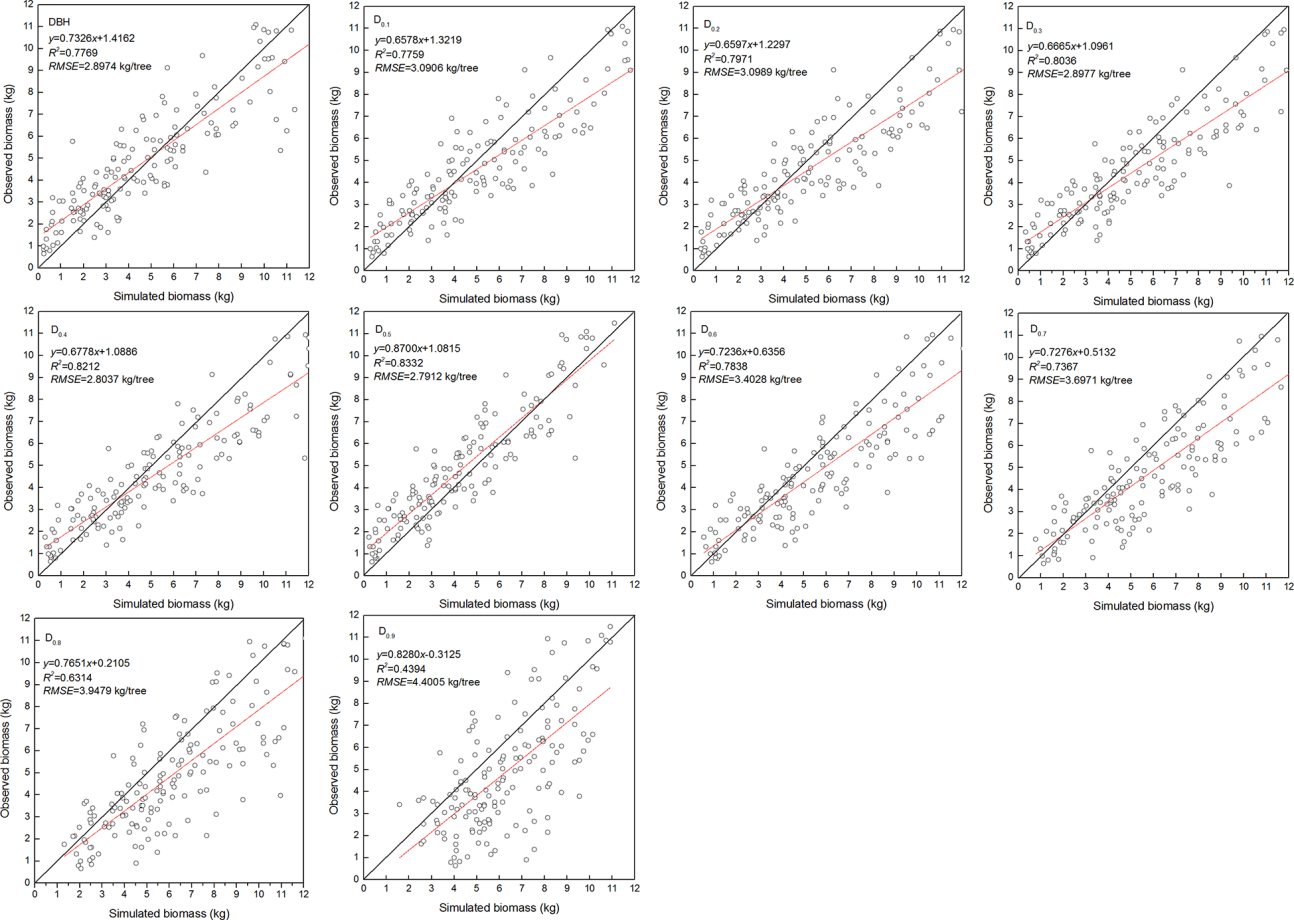
Linear relationship between observed and simulated data for foliage biomass. Diameter at breast height (DBH), diameter at relative tree height (D_i_, i = 0.1 to 0.9), simulated values (y), observed values (x), coefficient of determination (R^2^), and root mean square error (RMSE). The black and red line represents the 1:1 isoline and linear equation, respectively.

The estimated coefficients and the goodness-of-fit statistic of allometric biomass models with DBH or DRH as the predictor variable are shown in **Tables 4**–**5**. For the allometric growth relationship between the DRH (D_0.2_ and D_0.5_) for the stem and branch, the values of allometric exponent c were 2.6779 and 2.6754, respectively, which were the closest to the scaling relations predicted by the MST. For allometric growth relationship between the DRH (D_0.5_) and foliage biomass, the value of allometric exponent c was 2.3253, which was the closest to the scaling relations predicted by the geometric theory. However, when the independent variable was taken as DBH or DRH (D_0.1_, D_0.2_, D_0.3_, D_0.4_, and D_0.5_), the theoretical values (c=8/3) of the MST for stem and branch biomass models were included in the confidence interval (95%) of parameter estimation. The independent variable was taken as DBH or DRH (D_0.1_, D_0.2_, D_0.3_, D_0.4_, and D_0.5_), and the theoretical values (c=7/3) of the geometry theory for the foliage biomass model were included in the confidence interval of parameter estimation. When the allometric exponent c was most closely related to the theoretical value, the stem, branch, and foliage biomass allometric equations fit the best. The mean absolute bias (MAB) of stem, branch, and foliage allometric model were 17.074, 7.471, and 2.022, respectively. The mean percentage bias (MPB) of stem, branch, and foliage allometric model were 18.082, 7.859, and 2.103, respectively. The prediction accuracy of biomass allometric equations is shown in **Figures 2**–**4**. Simulated biomass overestimates observed biomass for branches and in case of foliage there is an underestimation for smaller values (**Figures 3** and **4**).

**Table 4:** Coefficient estimates for the biomass equations based on the DBH and diameter in relative tree height.

Organ	Variable	a	SE	c	SE
**Stem**	DBH	0.0408 (0.0122-0.0695)	0.0145	2.5433 (2.3497-2.7569)	0.1082
	D_0.1_	0.0479 (0.0199-0.0758)	0.0142	2.5632 (2.3809-2.7455)	0.0923
	**D_0.2_**	**0.0501 (0.0166-0.0844)**	**0.0172**	**2.6779 (2.4615-2.8044)**	**0.1122**
	D_0.3_	0.0513 (0.0168-0.0758)	0.0149	2.6125 (2.4382-2.8468)	0.1035
	D_0.4_	0.0819 (0.0342-0.1295)	0.0241	2.5635 (2.3965-2.7905)	0.0998
	D_0.5_	0.1277 (0.0522-0.2032)	0.0383	2.5432 (2.3345-2.7519)	0.1057
	D_0.6_	0.9539 (0.3967-1.5110)	0.2822	2.0887 (1.8514-2.3261)	0.1202
	D_0.7_	4.0336 (1.8760-6.1912)	1.0930	1.7305 (1.4761-1.9849)	0.1289
	D_0.8_	19.0987 (11.1440-27.0534)	4.0297	1.4103 (1.1141-1.7066)	0.1501
	D_0.9_	18.4907 (10.5960-26.3854)	3.9991	1.4380 (1.1327-1.7434)	0.1547
**Branch**	DBH	0.0046 (0.0002-0.0089)	0.0021	2.8158 (2.5322-3.0994)	0.1437
	D_0.1_	0.0052 (0.0009-0.0095)	0.0022	2.8523 (2.5958-3.1088)	0.1299
	D_0.2_	0.0053 (0.0004-0.0103)	0.0025	2.9203 (2.6266-3.2140)	0.1488
	D_0.3_	0.0068 (0.0001-0.0137)	0.0035	2.9148 (2.5872-3.1147)	0.1659
	D_0.4_	0.0116 (0.0013-0.0218)	0.0052	2.8169 (2.5191-3.1147)	0.1509
	**D_0.5_**	**0.0236 (0.0021-0.0450)**	**0.0109**	**2.6754 (2.3599-2.9989)**	**0.1619**
	D_0.6_	0.1070 (0.0148-0.1991)	0.0467	2.2452 (1.9252-2.5652)	0.1621
	D_0.7_	0.2993 (0.0435-0.5551)	0.1296	2.0201 (1.6719-2.3684)	0.1764
	D_0.8_	1.4041 (0.3917-2.4165)	0.5129	1.5947 (1.2490-1.9405)	0.1751
	D_0.9_	6.5179 (3.2284-9.8073)	1.6664	1.2140 (0.8473-1.5806)	0.1857
**Foliage**	DBH	0.0055 (0.0001-0.0110)	0.0028	2.3118 (2.0127-2.6109)	0.1515
	D_0.1_	0.0044 (0.0005-0.0083)	0.0020	2.4466 (2.1736-2.6596)	0.1383
	D_0.2_	0.0061 (0.0002-0.0120)	0.0030	2.4062 (2.0980-2.6543)	0.1561
	D_0.3_	0.0106 (0.0004-0.0213)	0.0054	2.2829 (1.9494-2.6164)	0.1689
	D_0.4_	0.0120 (0.0011-0.0229)	0.0055	2.3061 (1.9979-2.6143)	0.1561
	**D_0.5_**	**0.0116 (0.0022-0.0389)**	**0.0093**	**2.3253 (1.8922-2.5264)**	**0.1606**
	D_0.6_	0.0736 (0.0138-0.1334)	0.0303	1.8392 (1.5328-2.1457)	0.1552
	D_0.7_	0.1842 (0.0437-0.3247)	0.0712	1.6219 (1.3058-1.9381)	0.1602
	D_0.8_	0.6019 (0.2307-0.9731)	0.1880	1.3081 (1.0062-1.6100)	0.1529
	D_0.9_	2.2192 (1.2842-3.1543)	0.4736	0.9560 (0.6377-1.2743)	0.1612

DBH, diameter at breast height; SEs, approximate standard errors. The confidence intervals (95%) are in parentheses. The highlighted model parameters were used to estimate the biomass components.

**Table 5:** Goodness-of-fit statistics of regression biomass models in the cross-validation phase.

Variable	Stem			Branch			Foliage		
	MAB	RMSE	MPB	MAB	RMSE	MPB	MAB	RMSE	MPB
DBH	18.311	30.411	19.045	7.518	10.507	7.965	2.112	2.906	2.197
D_0.1_	17.593	27.263	18.298	10.782	15.679	11.214	2.128	3.109	2.214
D_0.2_	**17.074**	**26.966**	**18.082**	9.692	14.114	10.08	2.243	3.134	2.332
D_0.3_	18.571	30.819	19.315	7.821	11.001	8.283	2.097	2.905	2.181
D_0.4_	17.962	30.039	18.682	7.658	10.832	8.134	2.057	2.874	2.136
D_0.5_	17.671	27.561	18.378	**7.471**	**10.171**	**7.859**	**2.022**	**2.812**	**2.103**
D_0.6_	44.484	60.636	46.267	7.803	10.877	8.230	2.391	3.449	2.487
D_0.7_	63.874	77.612	66.433	7.913	11.268	8.770	2.648	3.713	2.754
D_0.8_	87.516	99.997	91.023	12.632	17.908	13.138	2.868	4.030	3.200
D_0.9_	154.925	172.866	161.132	15.075	20.454	15.679	3.076	4.497	3.469

The highlighted goodness-of-fit statistics were the best results of biomass components model fitting and evaluation.

### 3.3 Additive system of biomass components with DRH

**Figure 5:**
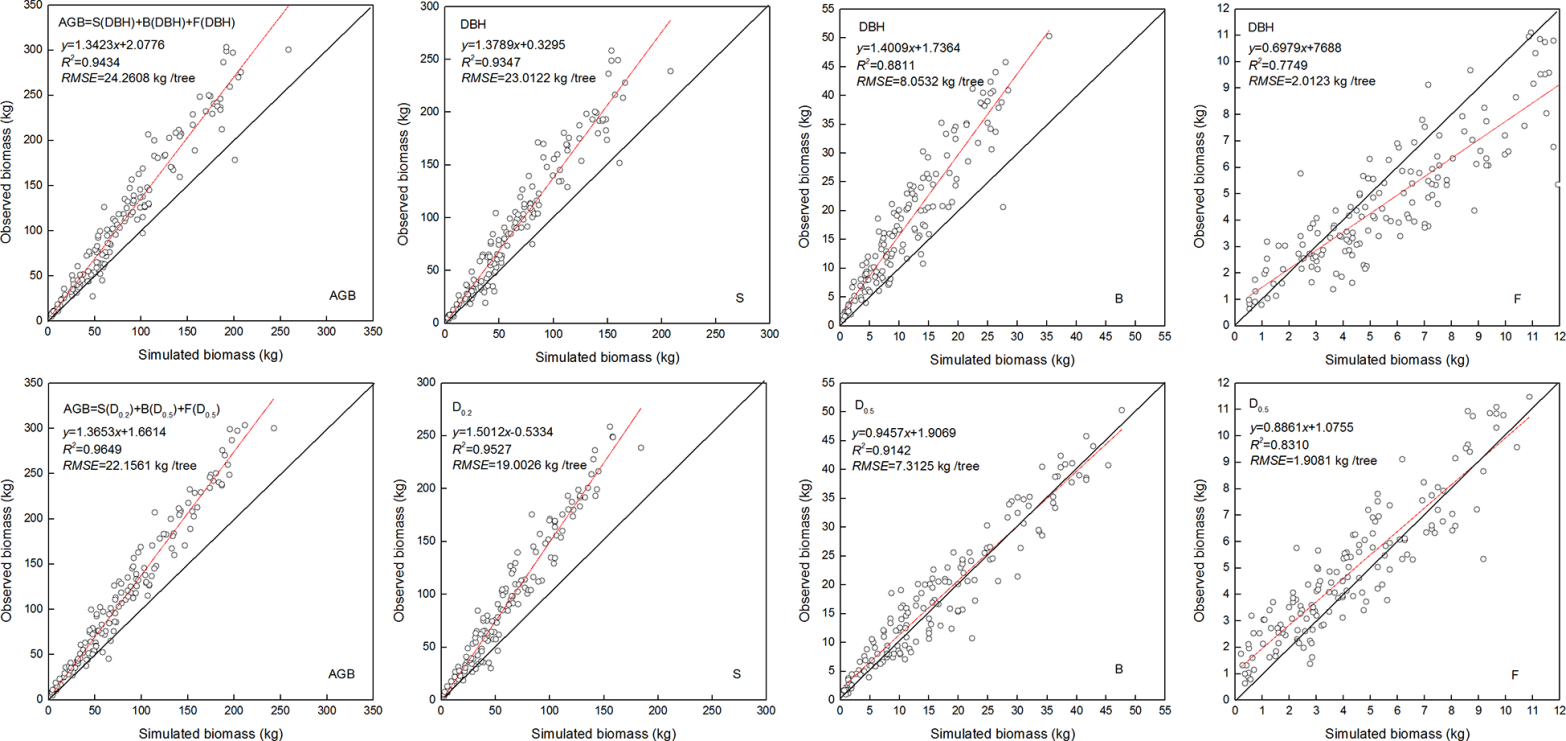
Linear relationship between observed and simulated data for the stem biomass (S), branch biomass (B), foliage biomass (F), and aboveground biomass (AGB). Diameter at breast height (DBH), diameter at relative tree height (D_i_, i = 0.2, 0.5, and 0.5) for stem, branch, and foliage, simulated values (y), observed values (x), coefficient of determination (R^2^), and root mean square error (RMSE). The black and red line represents the 1:1 isoline and linear equation, respectively.

The NSUR method was used to fit the additive systems of power exponent biomass equations to the biomass data. The estimated coefficients of the additive systems with DBH or DRH as the independent variable are shown in **Table 6**. As expected, the allometric exponent of the biomass components differed. Values that were estimated based on the DHR were closer to the theoretical value. Compared with the model fitted with DBH, the additive system with the DHR had smaller MAB, MPB, and RMSE for the total and component biomass (**Table 6**). Moreover, when the independent variable was the DHR, the observed and simulated values for stem biomass, branch biomass, foliage biomass, and aboveground biomass (AGB) were more similar. The prediction accuracy of biomass allometric equations is shown in **Figure 5**.

**Table 6:** Parameters and goodness-of-fit statistics for additive system of biomass equations by NSUR method.

Organ	Variables	a	SE	c	SE	MAB	RMSE	MPB
Stem	D_0.2_	0.0318	0.0081	2.6657	0.0809	13.0124	19.2234	15.4675
Branch	D_0.5_	0.0087	0.0037	2.6568	0.1363	5.3361	7.3604	5.5433
Foliage	D_0.5_	0.0127	0.1115	2.3151	0.0924	1.8276	2.1076	1.9455
AGB						17.0412	22.5687	20.3466
Stem	DBH	0.0508	0.0055	2.4518	0.0859	15.1542	23.0978	17.5211
Branch	DBH	0.0106	0.0165	2.3918	0.1288	6.2041	8.1436	6.4215
Foliage	DBH	0.0244	0.0183	1.8899	0.0885	1.9813	2.4492	2.0152
AGB						18.9534	24.3616	22.0551

SEs are approximate standard errors, AGB is the total tree aboveground biomass.

## 4 Discussion

### 4.1 Effects of DRH on process model

Although the DBH accounted for the most variation in the biomass models, a fixed position DBH leads to some deviation in the ability of the model to predict accurately, and the allometric exponent is not close to the theoretical values predicted because the tree size and stem shape are variable (Cienciala et al., 2013; Zianis et al., 2016). However, the DRH is not only the most powerful variable to explain the observed changes of tree biomass (Cienciala et al., 2013) but it can also fully verify the theory of allometric exponent. The biomass components models for the *L. principis-rupprechtii* plantation were constructed using the DBH and DRH, the estimated value of the parameters could be used to verify the theoretical model. The allometric exponent of the stem and branch was close to 8/3 and satisfied the MST when the independent variable was D_0.2_ and D_0.5_, respectively (**Table 5**). However, the allometric exponent of foliage was close to 7/3 using the diameter of relative tree height (D_0.5_), which satisfied the geometry theory (**Table 5**). In our study, the biomass of different organs could be highly accurately predicted when the allometric exponent was close to the theoretical value (**Figures 2** – **4**). Zianis et al. (2016) constructed stem biomass allometric equations for Hungarian oak (*Quercus frainetto* Ten.) based on the DBH, and the allometric exponent was not close to the theoretical values predicted. Nonlinear regression indicated that when the diameters at the relative height were d_0.1_ and d_0.5_, respectively (**Table 5**), the allometric exponent of stem was close to the theoretical value of MST for *Picea abies* and *Q. frainetto* (Fehrmann and Kleinn, 2006; Zianis and Radoglou, 2006). Zianis and Mencuccini (2004) and Cienciala et al. (2013) found that the allometric exponent was close to the value of the geometric theory and was significantly lower than the theoretical value of MST proposed by West et al (1999). In different regions and countries, the allometric exponent estimated by the fixed position diameter cannot come close to the theoretical values predicted owing to the growth characteristics of tree species, site quality (Eckmüller and Sterba, 2000; Grote and Reiter, 2004), and stand structure (Grote and Reiter, 2004).

In addition, we found that the allometric exponent of branch and foliage satisfied the MST and the geometry theory, respectively, when the independent variable was the diameter at the relative height (D_0.5_). We considered the possibility that pruning heights and crown structure could affect the allometric exponent of process model because the pruning height was in the range of one-half to one-third of the whole tree height for plantations in China. In addition, stand density and inter-tree competition could lead to the change of allometric exponent by affecting crown structure (Pothier et al., 2013; Forrester et al., 2021), while the diameter in relative tree height can reflect the effects of stand density, inter-tree competition and crown structure on the allometric exponent (Castle et al., 2017; Kumpu et al., 2020; Sharma, 2020). In our study, the biomass component model based on the DRH can not only meet the biological theory but also improve the accuracy of prediction of this model.

### 4.2 DRH prediction based on variable exponential taper equation

Several studies had demonstrated that the DRH was the most powerful variable to explain the observed changes in tree biomass (Cienciala et al., 2013; Zianis et al., 2016). However, the DRH is not as practical as that based on the DBH. Therefore, it highly critical to construct the taper equation to accurately predict the diameter of any position of trunk. To accurately estimate the diameter of any position at the stem, three forms of Kozak’s variable exponential taper equations were modeled, and the Kozak (2004) variable exponent taper equation was the optimal equation (**Table 3**). Some studies demonstrated that the Kozak (1988) and Kozak (2004) variable exponent taper equations could describe the shape of stem for some tree species (Corral-Rivas et al., 2007; Lumbres et al., 2017). However, the Kozak (2004)variable exponent taper equation is flexible and easily used (Xu et al., 2022). It has been proven to be applicable to many species in different countries (Huang et al., 2000; Klos et al., 2007; Jiang and Liu, 2011; He et al., 2021).

### 4.3 Effects of regression techniques and sample size

In this study, the variables of DBH and DRH to satisfy the theoretical scale parameters were used to develop the additive system of biomass models with the NSUR method (**Table 6**). Compared with the nonlinear regression, the estimated parameters of the biomass components estimated by NSUR were lower, and it was closer to the theoretical value and more precise at forecasting the values of *Larix principis-rupprechtii*. The system of additive models with the same explanatory variables (DBH) cannot accurately estimate the biomass of each component (Mohan et al., 2020). Indeed, the NSUR method is more suitable for developing additive systems of an allometric model based on the DRH because it can ensure the additivity properties of biomass components (Sanquetta et al., 2015; Dong et al., 2016). Considering the influence of biomass model structure and additive model construction on the accuracy of prediction, the usefulness and universality of biomass allometric theory parameters were substantially impacted by different regression methods (Sieg et al., 2009; Sileshi, 2014; Zianis et al., 2016). Compared with the NSUR method, the allometric exponent of biomass components estimated by nonlinear regression was higher for the *L. principis-rupprechtii* plantation. Similarly, some results showed that the allometric exponent estimated by nonlinear regression was larger than that of the reduced major axis regression and linear regression (Fehrmann and Kleinn, 2006; Hui et al., 2014; Zianis et al., 2016). However, this study found that the NSUR method was more accurate than nonlinear regression because it can effectively solve the additivity problem of the biomass equation through two or more different organ biomass equations (Parresol, 2001; Dong et al., 2018; Cao, 2021). For the given dataset, different regression techniques could affect the allometric parameters, and it was inevitable that a consistent conclusion could not be drawn (Sieg et al., 2009). Our results showed that applying the NSUR method with the DRH resulted in more accurate predictions than when the DBH was used (**Figure 5**), and the allometric exponent of biomass components was close to the theoretical values. With the DRH as the independent variable, the prediction accuracy of biomass model was improved by using NSUR method, but there was still some uncertainty (**Figure 5**). Building biomass models that increase crown variables may have higher accuracy (Forrester et al., 2021; Liang et al., 2022). In future research, DRH and crown variables can be combined to study the precision of biomass prediction model.

In addition to the regression technique, the sample size of individual tree biomass has a substantial influence on the allometric exponent (Bouriaud et al., 2019). The allometric exponent for the biomass components of *Larix principis-rupprechtii* conformed to the relationship identified for the whole compiled dataset (n=114). Small samples (<50 trees) of individual tree biomass result in problematic inferences of the theoretical value and confidence interval of allometric exponents (Coomes and Allen, 2009; Ebuy et al., 2011; Liu et al., 2019). However, large samples (≥50 trees) can explain more variation, which results in more efficient models and a reduction of variability in the allometric exponent (Zapata-Cuartas et al., 2012; Sileshi, 2014). Regression technology and sample size may affect the theoretical value of the allometric index of biomass components. Use of the NSUR method enabled the development of new additive systems of power exponent biomass equations using the DRH, which provided a reference for improving the accuracy of predicting biomass and carbon reserves in the future.

## 5 Conclusions

We developed the new additive system of biomass components by the diameter in relative tree height for a *Larix principis-rupprechtii* plantation. In this study, the Kozak (2004) variable exponential taper equation was developed to estimate the diameter at any point along the stem. Comparisons of the confidence intervals of different organs based on the DBH and DRH (nonlinear regression) indicated that the allometric exponent values were statistically similar (at 95% level) for the stems, branch, and foliage biomass models when the independent variable was DBH, D_0.1_, D_0.2_, D_0.3_, D_0.4_, or D_0.5_. In our study, the biomass of different organs could be highly accurately predicted when the allometric exponent was close to the theoretical value. Based on the DRH, the NSUR method were utilized to establish the biomass process model for the Larix principis-rupprechtii plantation, and the statistics for the NSUR fit better than nonlinear regression, and the scaling factor c was very close to the theoretical value. For the allometric relation between DRH (D_0.2_ and D_0.5_) for the stems and branches, the value of scaling factor c was the closest to scaling relations predicted by the MST. For the allometric relation between DRH (D_0.5_) and foliage biomass, the value of scaling factor c was the most closely related to the scaling relations predicted by the geometric theory. The new additive system of biomass process models based on the DRH is advantageous owing to its theory, high precision, and popularization, which provides a strong support for the biomass and carbon sink evaluation of *Larix principis-rupprechtii* plantations in north China.

## Data availability statement

The original contributions presented in the study are included in the article/supplementary materials. Further inquiries can be directed to the corresponding authors.

## Author contributions

DW and ZZ contributed to the study design and performed the formal analysis. DW performed the software analysis and wrote the first draft of the manuscript. DZ contributed data curation, and ZZ and XH contributed to the writing, review and editing. All authors contributed to the article and approved the submitted version.
